# LCP crystallization and X-ray diffraction analysis of VcmN, a MATE transporter from *Vibrio cholerae*

**DOI:** 10.1107/S2053230X16008931

**Published:** 2016-06-22

**Authors:** Tsukasa Kusakizako, Yoshiki Tanaka, Christopher J. Hipolito, Teruo Kuroda, Ryuichiro Ishitani, Hiroaki Suga, Osamu Nureki

**Affiliations:** a Department of Biological Sciences, Graduate School of Science, The University of Tokyo, 2-11-16 Yayoi, Bunkyo-ku, Tokyo 113-0032, Japan; b Department of Systems Biology, Graduate School of Biological Sciences, Nara Institute of Science and Technology, 8916-5 Takayama-cho, Ikoma, Nara 630-0192, Japan; c Department of Chemistry, Graduate School of Science, The University of Tokyo, 7-3-1 Hongo, Bunkyo-ku, Tokyo 113-0033, Japan; d Faculty of Medicine, University of Tsukuba, 1-1-1 Tennodai, Tsukuba 305-8575, Japan; e Department of Molecular Microbiology and Biotechnology, Graduate School of Biomedical and Health Sciences, Hiroshima University, 1-2-3 Kasumi, Minami-ku, Hiroshima 734-8553, Japan

**Keywords:** MATE transporter, multidrug resistance, lipidic cubic phase, LCP

## Abstract

Multidrug and toxic compound extrusion (MATE) transporters, one of the multidrug exporter families, efflux xenobiotics towards the extracellular side of the membrane. Since MATE transporters expressed in bacterial pathogens contribute to multidrug resistance, they are important therapeutic targets. Here, a MATE-transporter homologue from *Vibrio cholerae*, VcmN, was overexpressed in *Escherichia coli*, purified and crystallized in lipidic cubic phase (LCP). X-ray diffraction data were collected to 2.5 Å resolution from a single crystal obtained in a sandwich plate. The crystal belonged to space group *P*2_1_2_1_2_1_, with unit-cell parameters *a* = 52.3, *b* = 93.7, *c* = 100.2 Å. As a result of further LCP crystallization trials, crystals of larger size were obtained using sitting-drop plates. X-ray diffraction data were collected to 2.2 Å resolution from a single crystal obtained in a sitting-drop plate. The crystal belonged to space group *P*2_1_2_1_2_1_, with unit-cell parameters *a* = 61.9, *b* = 91.8, *c* = 100.9 Å. The present work provides valuable insights into the atomic resolution structure determination of membrane transporters.

## Abstract 2

A *V. cholerae* MATE transporter was crystallized using the lipidic cubic phase (LCP) method. X-ray diffraction data sets were collected from single crystals obtained in a sandwich plate and a sitting-drop plate to resolutions of 2.5 and 2.2 Å, respectively.

## 1. Introduction   

The increase in multidrug-resistant pathogens that are difficult to treat using conventional antibiotics has become a global public health problem (Reimer *et al.*, 2011 ▸; Miwanda *et al.*, 2015 ▸). One of the multidrug-resistance mechanisms is to actively export the invading xenobiotics using multidrug-resistance (MDR) transporters (Blair *et al.*, 2014 ▸). MDR transporters are classified into at least five families, including the ATP-binding cassette (ABC) superfamily, the resistance–nodulation–division (RND) family, the major facilitator superfamily (MFS), the small multidrug-resistance (SMR) family and the multidrug and toxic compound extrusion (MATE) family (Du *et al.*, 2015 ▸). MATE transporters are secondary active transporters that use the electrochemical energy of an Na^+^ or H^+^ gradient across the membrane (Morita *et al.*, 2000 ▸; He *et al.*, 2004 ▸; Jin *et al.*, 2014 ▸) and are conserved among bacteria, archaea and eukarya (Omote *et al.*, 2006 ▸). They efflux chemically and structurally distinct organic cations, such as fluoroquinolones (*e.g.* norfloxacin) and fluorescent dyes (*e.g.* Hoechst 33342). Since MATE transporters from bacterial pathogens contribute to their multidrug resistance (Kaatz *et al.*, 2005 ▸; McAleese *et al.*, 2005 ▸), they are potential therapeutic targets. To date, the crystal structures of five prokaryotic MATE-transporter homologues have been reported (He *et al.*, 2010 ▸; Lu, Symersky *et al.*, 2013 ▸; Tanaka *et al.*, 2013 ▸; Lu, Radchenko *et al.*, 2013 ▸; Radchenko *et al.*, 2015 ▸; Mousa *et al.*, 2016 ▸). The structures have provided insight into substrate recognition and transport by MATE transporters. MATE transporters are composed of 12 transmembrane (TM) helices, which are divided into the N-lobe (TM1–TM6) and the C-lobe (TM7–TM12). A cleft is formed between the N- and C-lobes, and is divided into the N- and C-lobe cavities. This central cleft is opened towards the extracellular side in all of the reported structures. The structures of complexes with substrates demonstrated that the substrates bind to the N-lobe cavity (Tanaka *et al.*, 2013 ▸; Lu, Radchenko *et al.*, 2013 ▸). Furthermore, the inhibitor-bound structures revealed that inhibitors partially occupy the substrate-binding site, resulting in inhibition of the substrate-transport activity by MATE transporters (Tanaka *et al.*, 2013 ▸; Radchenko *et al.*, 2015 ▸). However, the binding sites of substrates and inhibitors are diverse among the MATE-transporter homologues, and the amino-acid residues involved in the recognition of substrates and inhibitors are not conserved. For the development of effective inhibitors against MATE transporters, structural analyses of MATE-transporter homologues from clinically relevant multidrug-resistant pathogens, such as *Staphylococcus aureus* and *Vibrio cholerae*, are needed. To date, six MATE-transporter homologues from *V. cholerae* have been identified (Brown *et al.*, 1999 ▸; Huda *et al.*, 2001 ▸, 2003 ▸; Begum *et al.*, 2005 ▸); however, of these homologues, only medium-resolution structures of NorM-VC (∼3.65 Å) have been reported (He *et al.*, 2010 ▸). Here, we performed a structural analysis of one of the *V. cholerae* MATE-transporter homologues, VcmN, which shares approximately 17% sequence identity with NorM-VC. We report the expression, purification, lipidic cubic phase (LCP) crystallization and X-ray diffraction analysis of VcmN. Moreover, we describe its co-crystallization with macrocyclic peptides and a substrate-soaking experiment with the aim of determinating complex structures. Since there is little literature on the practical LCP crystallization method for membrane transporters, our study provides valuable insights into crystallization techniques for the growth of crystals of membrane transporters that diffract to high resolution.

## 2. Materials and methods   

### 2.1. Macromolecule production   

The gene encoding full-length VcmN (UniProt ID C3LWQ2) was subcloned into a modified pET-28a vector. This modified pET-28a vector contains a C-terminal *Tobacco etch virus* (TEV) protease cleavage site (ENLYFQG) followed by a His_6_ tag. The ten C-terminal residues of full-length VcmN and the two vector-derived residues were truncated by a PCR-based method using PrimeSTAR Max DNA polymerase (Takara Bio). The resulting construct was designated VcmNΔC. The plasmid was introduced into *Escherichia coli* C41(DE3) Rosetta cells harbouring pRARE, which encodes the tRNAs for codons rarely used in *E. coli*. The typical purification method is described below. The transformed cells were grown in 2.5 l Luria–Bertani (LB) medium containing 30 µg ml^−1^ kanamycin at 310 K. When the absorbance at 600 nm (*A*_600_) reached 0.5–0.8, expression was induced with 0.4 m*M* isopropyl β-d-1-thiogalactopyranoside (IPTG) and the cells were grown for about 20 h at 293 K. The cells were centrifuged at 5000*g* for 10 min and disrupted by 2–3 passes at 103 MPa using a Microfluidizer (Microfluidics). After centrifugation to remove debris at 28 000*g* for 30 min, the supernatant was ultracentrifuged at 125 000*g* for 1 h to collect the membrane fraction. The membrane fraction was resuspended in 20 m*M* Tris–HCl pH 8.0, 300 m*M* NaCl and stored at 193 K until use. The membrane fraction was solubilized in 20 m*M* Tris–HCl pH 8.0, 300 m*M* NaCl, 20 m*M* imidazole, 1.5% *n*-dodecyl-β-d-maltoside (DDM) for 1 h at 277 K. After the removal of debris by ultracentrifugation at 125 000*g* for 30 min, the supernatant was mixed with 5 ml Ni–NTA resin (Qiagen) equilibrated with buffer *A* (20 m*M* Tris–HCl pH 8.0, 300 m*M* NaCl, 0.1% DDM) containing 20 m*M* imidazole for about 1 h at 277 K. The mixture was loaded into an Econo-column (Bio-Rad) and the flowthrough fraction was discarded. The resin was washed with ten column volumes of buffer *A* containing 30 m*M* imidazole and the protein sample was eluted with buffer *A* containing 300 m*M* imidazole. To cleave the His_6_ tag, His-tagged TEV protease (produced in-house) was added to the eluted fraction in a 10:1(*w*:*w*) protein:TEV protease ratio. During the overnight cleavage reaction at 277 K, the solution was dialyzed against 20 m*M* Tris–HCl pH 8.0, 300 m*M* NaCl, 0.02% DDM. The solution was mixed with Ni–NTA resin again for 1 h at 277 K to remove the TEV protease. The flowthrough fraction was concentrated using an Amicon Ultra centrifugal filter (50 kDa molecular-weight cutoff, Millipore) and applied onto a Superdex 200 Increase (GE Healthcare) column equilibrated with 20 m*M* Tris–HCl pH 8.0, 100 m*M* NaCl, 0.1% DDM. The peak fractions were concentrated to approximately 10 mg ml^−1^ using an Amicon Ultra filter (50 kDa molecular-weight cutoff, Millipore). To remove the Na^+^ ions, a portion of the sample was dialyzed against 20 m*M* Tris–HCl pH 8.0, 0.02% DDM. The purity of the protein sample was assessed by SDS–PAGE. The macromolecule-production information is summarized in Table 1 ▸.

### 2.2. Crystallization   

Previously, co-crystallization with macrocyclic peptides improved the quality of crystals of selenomethionine-labelled *Pyrococcus furiosus* MATE (*Pf*MATE), thereby facilitating its structure determination. Moreover, the macrocyclic peptides showed inhibitory activity against *Pf*MATE (Tanaka *et al.*, 2013 ▸; Hipolito *et al.*, 2013 ▸). Thus, to improve the quality of the VcmN crystals, an *in vitro* selection of macrocyclic peptides that bind to VcmN was also performed using the random nonstandard peptide integrated discovery (RaPID) system (Hipolito & Suga, 2012 ▸). Crystallization was performed using the LCP method (Caffrey & Cherezov, 2009 ▸), as the structure of *Pf*MATE was also determined at high resolution using this crystallization method (Tanaka *et al.*, 2013 ▸). The protein solution containing Na^+^ was mixed with 8 m*M* macrocyclic peptides dissolved in dimethyl sulfoxide in a 10:1(*v*:*v*) ratio, and the mixture was incubated for 1 h at 277 K. The sample was mixed with monoolein (Nu-Chek Prep) in a 2:3(*w*:*w*) protein:lipid ratio using coupled syringes. Drops of the mixture (50 nl) were dispensed onto a 96-well plastic sandwich plate (SWISSCI) and were overlaid with 600 nl reservoir solution using a Mosquito LCP crystallization robot (TTP Labtech). Initial crystallization screening was performed at 293 K using the MemMeso crystallization kit (Molecular Dimensions) and in-house-produced grid-screening crystallization kits as reservoir solutions. For crystallization optimization, StockOptions Salt and Additive Screen (Hampton Research) were added to the reservoir solutions, in addition to optimization of the pH and the concentrations of precipitants and salts. Furthermore, 40 nl portions of a mixture of protein samples without Na^+^ and monoolein in a 2:3(*w*:*w*) ratio were spotted on a 96-well sitting-drop plate (AS ONE) and overlaid with 800 nl reservoir solution. The obtained crystals were overlaid with 800 nl reservoir solution containing 10 m*M* Hoechst 33342 as a substrate (Begum *et al.*, 2005 ▸) and were incubated at 293 K. The crystals were picked up using MicroMounts (MiTeGen) or LithoLoops (Protein Wave) and were flash-cooled in liquid nitrogen. Crystallization information is provided in Table 2 ▸.

### 2.3. Data collection and processing   

All X-ray diffraction data sets were collected by the helical data-collection method using a micro-focus beam at SPring-8 beamline BL32XU (Hirata *et al.*, 2013 ▸). Data processing was performed using *XDS* (Kabsch, 2010 ▸), *DIALS* (Waterman *et al.*, 2013 ▸) and *AIMLESS* (Evans & Murshudov, 2013 ▸). Molecular replacement was performed with *Phaser* (McCoy *et al.*, 2007 ▸) using the structure of *Pf*MATE (PDB entry 3vvn; Tanaka *et al.*, 2013 ▸) as the search model. Model building and refinement were performed with *Coot* (Emsley & Cowtan, 2004 ▸; Emsley *et al.*, 2010 ▸) and *phenix.refine* (Adams *et al.*, 2010 ▸; Afonine *et al.*, 2012 ▸), respectively. The data-collection statistics are summarized in Table 3 ▸.

## 3. Results and discussion   

**Figure 1:**
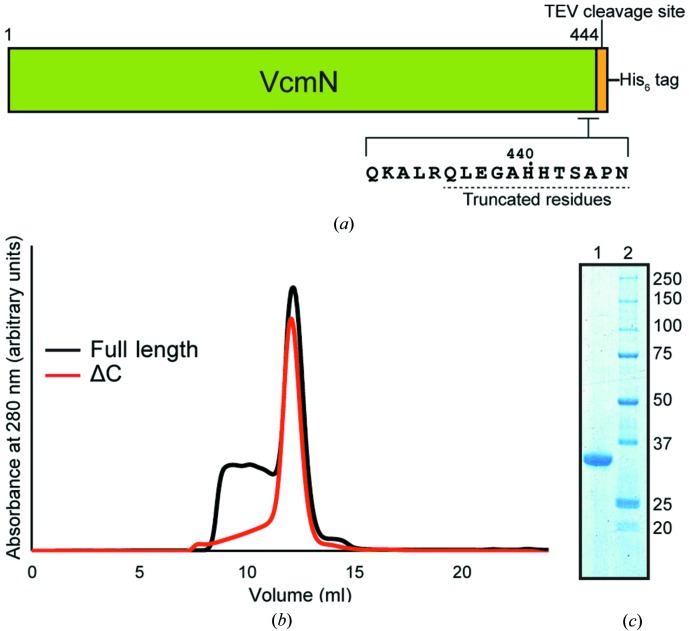
Protein preparation. (*a*) Schematic diagram of the VcmN crystallization construct. (*b*) Chromatograms of full-length VcmN and the VcmNΔC construct. (*c*) SDS–PAGE analysis with Coomassie Brilliant Blue staining. Lane 1, VcmNΔC; lane 2, molecular-weight markers (labelled in kDa).

In general, the expression level and stability of the protein sample are important factors for structural analysis. We first overexpressed and purified full-length VcmN. However, the full-length VcmN partially precipitated during the concentration step. In addition, the size-exclusion chromatography (SEC) chromatogram peak was not monodisperse, thus indicating instability of full-length VcmN. To improve the stability, the C-terminal 12 residues of full-length VcmN were truncated as the C-terminal residues of VcmN were predicted to be disordered based on a *DISOPRED*2 analysis (Ward *et al.*, 2004 ▸). During the purification of VcmNΔC, the loss of sample by aggregation was suppressed compared with that of the full-length protein, and the SEC chromatogram peak showed monodispersity. The final yield increased from 1 to 5 mg per 2.5 l culture on truncating the C-terminal 12 residues. The schematic diagram of the constructs, the SEC chromatograms and the Coomassie Brilliant Blue-stained gel are shown in Fig. 1 ▸.

**Figure 2:**
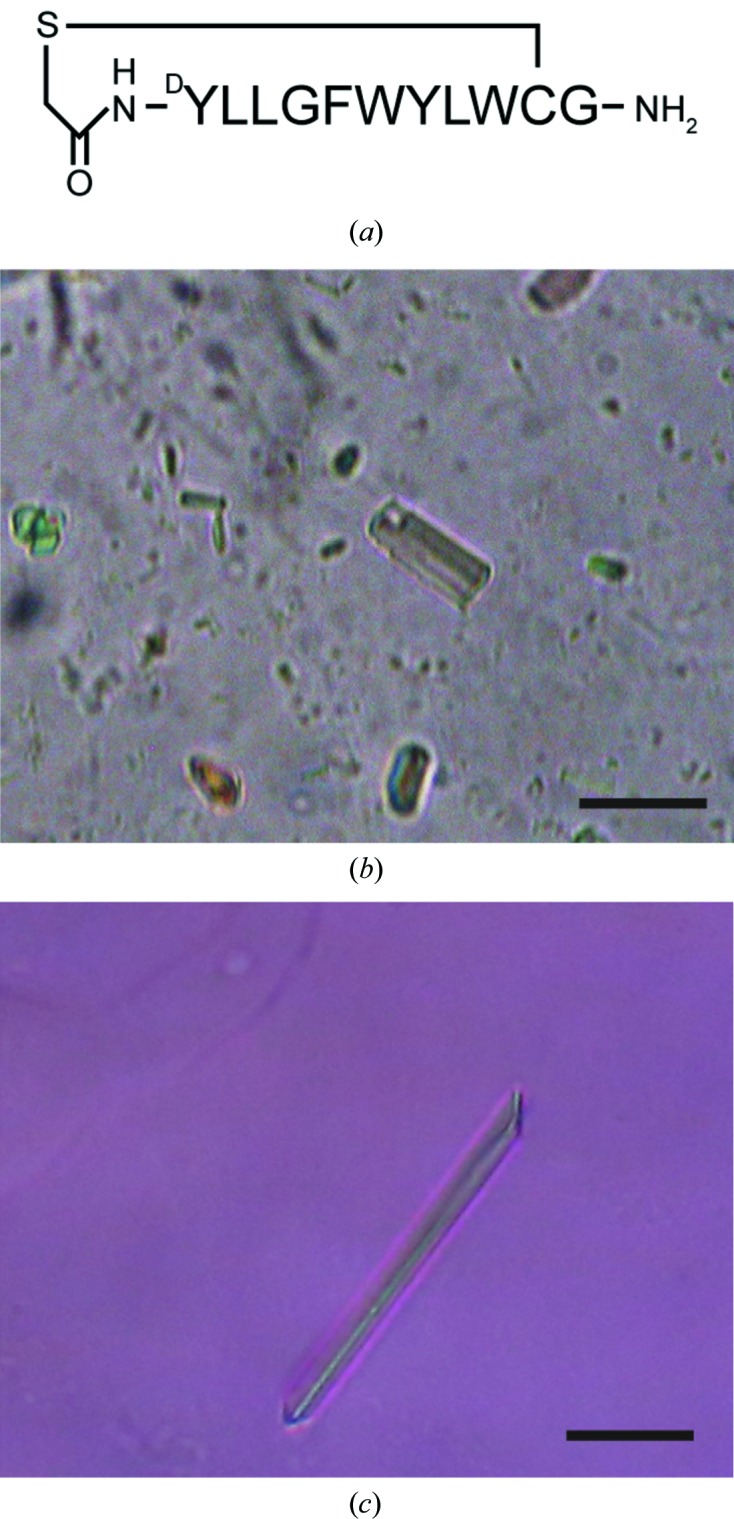
Crystals of VcmNΔC. (*a*) Schematic representation of the macrocyclic peptide. (*b*) Form *A* crystal. (*c*) Form *B* crystal. The scale bars represent 30 µm.

We performed an LCP crystallization screening of VcmNΔC. Initially, tiny crystals of a mixture of VcmNΔC and the macrocyclic peptide (Fig. 2 ▸ *a*) were obtained under several conditions containing precipitants such as polyethylene glycol (PEG) 300 and PEG 400 using sandwich plates. As a result of optimization efforts, including the screening of additives using the StockOptions Salt and Additive Screen kits, rectangular prism-shaped crystals (form *A*) were obtained with a reservoir solution composed of 30% PEG 300, 100 m*M* sodium citrate pH 5.0, 100 m*M* ammonium fluoride (Fig. 2 ▸ *b*). Crystals appeared in a day and grew to approximate dimensions of 10 × 10 × 25 µm in a week. We also attempted crystallization of VcmNΔC without the macrocyclic peptides. In contrast to the crystallization of *Pf*MATE, we unfortunately found that the peptides did not affect the quality of the VcmNΔC crystals. Next, crystallization screening was performed using sitting-drop plates. In contrast to crystallization in sandwich plates, crystallization in sitting-drop plates is suitable for substrate-soaking experiments to determine the structures of complexes with substrates. Rod-shaped crystals (form *B*) were obtained in a reservoir composed of 30% PEG 500 dimethyl ether (DME), 100 m*M* Tris–HCl pH 8.0, 100 m*M* magnesium formate. The approximate dimensions of the form *B* crystals were 10 × 10 × 50 µm. The sizes of the crystals obtained in the sitting-drop plates were usually larger than those produced in the sandwich plates. Finally, as a result of optimization of the salt concentration and the pH of the condition, crystals with approximate dimensions of 10 × 10 × 100 µm were obtained in reservoir solutions composed of 28–33% PEG 500 DME, 100 m*M* Tris–HCl pH 7.5, 50 m*M* magnesium formate (Fig. 2 ▸ *c*). For the substrate-soaking experiments, an 800 nl portion of reservoir solution containing 10 m*M* Hoechst 33342 was added to drops containing crystals. The protein crystals were incubated for about one month at 293 K and then cooled in liquid nitrogen.

**Figure 3:**
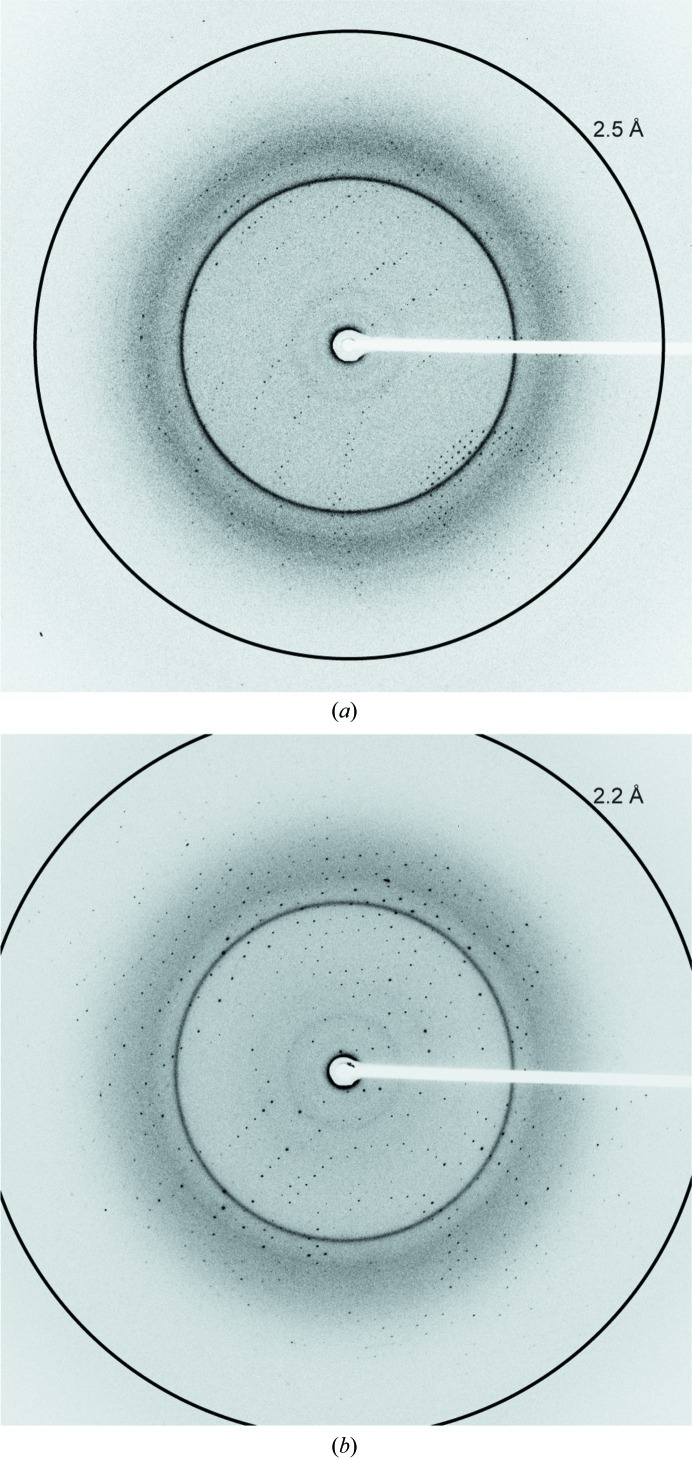
X-ray diffraction images of the form *A* (*a*) and form *B* (*b*) crystals. The rings indicate 2.5 Å (*a*) and 2.2 Å (*b*) resolution, respectively.

A form *A* single crystal diffracted X-rays to 2.5 Å resolution (Fig. 3 ▸ *a*) and belonged to space group *P*2_1_2_1_2_1_, with unit-cell parameters *a* = 52.3, *b* = 93.7, *c* = 100.2 Å. The calculated Matthews coefficient is 2.58 Å^3^ Da^−1^ with 52.4% solvent content, assuming the presence of one molecule in the asymmetric unit. The form *B* single crystal diffracted X-rays to 2.2 Å resolution (Fig. 3 ▸ *b*) and belonged to space group *P*2_1_2_1_2_1_, with unit-cell parameters *a* = 61.9, *b* = 91.8, *c* = 100.9 Å. The calculated Matthews coefficient is 3.01 Å^3^ Da^−1^ with 59.2% solvent content, assuming the presence of one molecule in the asymmetric unit. Molecular replacement was performed using the structure of *Pf*MATE as the search model and an interpretable electron-density map was obtained. However, we have not yet observed an electron-density peak corresponding to either the macrocyclic peptide or Hoechst 33342. Model building and structure refinement are under way.

## Figures and Tables

**Table 1:** Macromolecule-production information Cloning artifacts are underlined. The truncated residues are in parentheses.

Source organism	*V. cholerae*
DNA source	UniProt ID C3LWQ2
Expression vector	Modified pET-28a
Expression host	*E. coli* strain C41(DE3) Rosetta
Complete amino-acid sequence of the construct produced	MAMQTSTSSLAKQLFQMTWPMLFGVLSLMSFQLVDSAFIGQLGVLPLAAQGFTMPIQMVIIGIQVGLGIATTAVISRAIGAGKTEYAKQLGGLVIVIGGIGVALIALVLYLLRQPLLGLLGAPETVFAIIDHYWLWWLASAWTGAMLYFYYSVCRANGNTLLPGTLMMVTSVLNLILDPIFIFTFDLGIDGAAIATIIAFGVGIAIVAPKVAQRQWTSYQWQDLNISQSLTALGHIMGPAMLSQLLPPLSSMFATKLLASFGTAAVAAWALGSRFEFFALVAVLAMTMSLPPMIGRMLGAKEITHIRQLVRIACQFVLGFQLLIALVTYVFATPLAELMTSETEVSQILNLHLVIVPISLGALGICMLMVSVANALGKSYVALTISALRLFAFYLPCLWLGAHFYGIEGLFIGALVGNIIAGWAAWLAYQKALR(QLEGAHHTSAPN)SENLYFQGQVDKLAAALEHHHHHH

**Table 2:** Crystallization

	Form *A* crystal	Form *B* crystal
Method	Lipidic cubic phase	Lipidic cubic phase
Plate type	96-well plastic sandwich plate	96-well sitting-drop plate
Temperature (K)	293	293
Protein concentration (mg ml^−1^)	10	10
Buffer composition of protein solution	20 m*M* Tris–HCl pH 8.0, 100 m*M* NaCl, 0.1% DDM	20 m*M* Tris–HCl pH 8.0, 0.02% DDM
Co-crystallized compound (final concentration in mixture with protein solution)	0.8 m*M* macrocyclic peptide	—
Composition of reservoir solution	30% PEG 300, 100 m*M* sodium citrate pH 5.0, 100 m*M* ammonium fluoride	28–33% PEG 500 DME, 100 m*M* Tris–HCl pH 7.5–8.0, 50–100 m*M* magnesium formate
Volume of LCP drop (nl)	50	40
Volume of reservoir (nl)	600	800
Soaked compound (final concentration in crystallization drop)	—	5 m*M* Hoechst 33342

**Table 3:** Data collection and processing Values in parentheses are for the outer shell.

	Form *A* crystal	Form *B* crystal
Diffraction source	BL32XU, SPring-8	BL32XU, SPring-8
Wavelength (Å)	1.0	1.0
Temperature (K)	100	100
Detector	Rayonix MX225HE	Rayonix MX225HS
Crystal-to-detector distance (mm)	240	240
Rotation range per image (°)	1.0	1.0
Total rotation range (°)	180	178
Exposure time per image (s)	1.0	1.0
Space group	*P*2_1_2_1_2_1_	*P*2_1_2_1_2_1_
*a*, *b*, *c* (Å)	52.3, 93.7, 100.2	61.9, 91.8, 100.9
Resolution range (Å)	93.74–2.50 (2.60–2.50)	45.91–2.20 (2.26–2.20)
Total No. of reflections	118705	210722
No. of unique reflections	17737	29974
Completeness (%)	99.8 (98.5)	99.8 (97.2)
Multiplicity	6.7 (5.9)	7.0 (5.0)
〈*I*/σ(*I*)〉	5.9 (1.9)	7.8 (1.2)
*R*_meas_	0.252 (0.995)	0.208 (1.611)
CC_1/2_	0.989 (0.635)	0.995 (0.477)
Overall *B* factor from Wilson plot (Å^2^)	31.7	24.3
